# Frontier of Self and Impact Prediction

**DOI:** 10.3389/fpsyg.2018.01073

**Published:** 2018-06-27

**Authors:** Justine Cléry, Suliann Ben Hamed

**Affiliations:** UMR5229, Institut des Sciences Cognitives Marc Jeannerod, CNRS-Université Claude Bernard Lyon I, Bron, France

**Keywords:** visual, tactile, looming stimuli, prediction, multisensory integration, peripersonal space

## Abstract

The construction of a coherent representation of our body and the mapping of the space immediately surrounding it are of the highest ecological importance. This space has at least three specificities: it is a space where actions are planned in order to interact with our environment; it is a space that contributes to the experience of self and self-boundaries, through tactile processing and multisensory interactions; last, it is a space that contributes to the experience of body integrity against external events. In the last decades, numerous studies have been interested in peripersonal space (PPS), defined as the space directly surrounding us and which we can interact with (for reviews, see Cléry et al., 2015b; de Vignemont and Iannetti, 2015; di Pellegrino and Làdavas, 2015). These studies have contributed to the understanding of how this space is constructed, encoded and modulated. The majority of these studies focused on subparts of PPS (the hand, the face or the trunk) and very few of them investigated the interaction between PPS subparts. In the present review, we summarize the latest advances in this research and we discuss the new perspectives that are set forth for futures investigations on this topic. We describe the most recent methods used to estimate PPS boundaries by the means of dynamic stimuli. We then highlight how impact prediction and approaching stimuli modulate this space by social, emotional and action-related components involving principally a parieto-frontal network. In a next step, we review evidence that there is not a unique representation of PPS but at least three sub-sections (hand, face and trunk PPS). Last, we discuss how these subspaces interact, and we question whether and how bodily self-consciousness (BSC) is functionally and behaviorally linked to PPS.

## Peripersonal Space

In everyday life, we are solicited by multiple stimuli in our environment. The space around us is filled with conspecifics, animals and objects, often animated by their own goals. Most of the time, this implies interacting with these elements of the environment along a very rich and complex repertoire that depends on the context and the very nature of this environment. This requires the construction of a coherent representation of our body and the selective encoding of the space immediately surrounding it, the so-called peripersonal space (PPS), both in order to estimate the consequences of the environment and the consequences of our own actions onto our body. Interestingly, the PPS is subserved in the brain by specific neuronal mechanisms embedded in a well identified cortical network that specifically processes visual or auditory information occurring in the space that directly surrounds us as well as the tactile information occurring on the body.

### Visuo-Tactile Neurons as a Substrate for PPS Encoding in the Cortex

**FIGURE 1:**
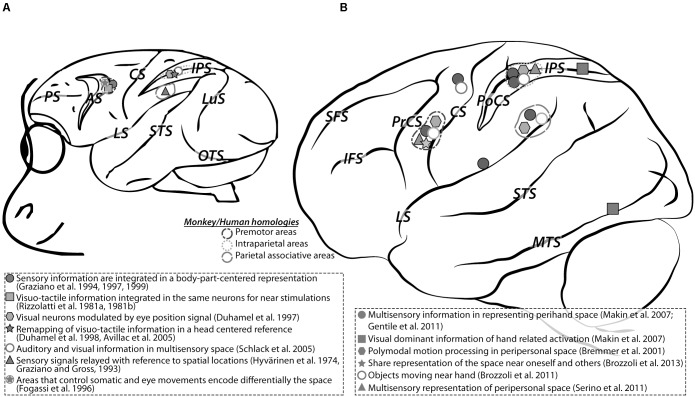
Functional regions involved in peripersonal space coding in monkeys **(A)** and in humans **(B)**. Three homologous regions coding peripersonal space representation have been found in monkeys and humans: premotor, intraparietal and parietal associative areas. *Cortical sulci: AS, arcuate sulcus; CS, central sulcus; IFS, inferior frontal sulcus; IPS, intraparietal sulcus; LS, lateral sulcus; LuS, luneate sulcus; MTS, middle temporal sulcus; PoCS, postcentral sulcus; PrCS, precentral sulcus; PS, principal sulcus; SFS, superior frontal sulcus; STS, superior temporal sulcus; OTS, occipito-temporal sulcus.*

Numerous studies in non-human primates have shown that multisensory cues, and specifically those recruiting the body through touch, are integrated by a specialized neural system representing PPS (**Figure 1A**). While much of the work has focused on visuo-tactile interactions, audio-tactile properties of PPS have also been explored. Specific populations of multisensory neurons respond both to tactile information on the body (arm, face or trunk) and visual or auditory stimuli occurring in PPS, i.e., close to the body. These multisensory neurons have first been described in the macaque brain, in a network composed by specialized parietal and frontal areas: the ventral premotor cortex (vPM; F4, Rizzolatti et al., 1981a,b; or polysensory zone PZ, Graziano et al., 1994, 1997, 1999; Fogassi et al., 1996; Graziano and Cooke, 2006; Guipponi et al., 2015), the ventral intraparietal area on the fundus of the intraparietal sulcus (VIP, Hyvärinen and Poranen, 1974; Duhamel et al., 1997, 1998; Avillac et al., 2005; Schlack et al., 2005; Graziano and Cooke, 2006; Guipponi et al., 2013, 2015), in the parietal areas 7b as well as in subcortical regions such as the putamen (Graziano and Gross, 1993). Though the response properties of these neurons are modulated by eye position their visual receptive fields (RFs) are anchored to specific body parts. This suggests that the multisensory representation of PPS they hold, is body-part centered, for example on the head for area VIP neurons (Duhamel et al., 1997; Avillac et al., 2005) or on the arm for premotor PZ neurons (Graziano et al., 2000; Graziano and Cooke, 2006). While these studies point toward a functional convergence between PPS processing and multisensory convergence processes, very few of them have explicitly probed that these multisensory neurons actively integrate sensory information from different modalities (Avillac et al., 2007), and even fewer have explicitly probed a direct link between multisensory visuo-tactile or audio-tactile integration and PPS processing. In a recent study performed in epileptic patients, Bernasconi et al. (2018) recorded for the first time surface intracranial electroencephalography signals (ECoG) while tactile and/or approaching auditory stimulations are presented to the subjects. The authors show that PPS processing most often coincides with multisensory integration processes.

### Clinical Evidence for Visuo-Tactile Interactions in PPS

Extinction is a neurological condition in which patients fail to detect contralesional stimuli only when challenged in their sensory processing by the presentation of a double simultaneous stimulation, both on the ipsilesional and contralesional sides (Bender, 1952; Mattingley et al., 1997; Làdavas and Serino, 2008). This condition is observed both when the concurrent stimuli are from the same sensory modality (e.g., both visual, this condition is referred to as unimodal extinction) and when the concurrent stimuli are from two different modalities (e.g., one is visual and the other is tactile, this condition is referred to as cross-modal extinction). In such right brain-damaged patients with tactile extinction, visual or auditory stimulations on the ipsilesional side exacerbate contralesional tactile extinction. In contrast, if the visual and tactile stimuli are both presented on the same contralesional side, then, the clinical deficit is reduced, the processing of one sensory stimulus benefiting from the processing of the other one (Làdavas et al., 1998a). Therefore, cross-modal extinction depends on the spatial arrangement of the stimuli relative to the patient’s body (Farnè et al., 2005a,b; for review, see Làdavas, 2002). Importantly, this modulation is most systematic when visuo-tactile interactions occur in the space near to the patients’ body, as compared to the space far away (di Pellegrino et al., 1997; Làdavas et al., 1998a, 2000). This finding is taken as evidence for the existence of a PPS in the human brain, relying on the integration of visual and tactile information in the space close to the body, in a way very similar to that described in monkeys (Làdavas, 2002). Most of these studies place the bimodal stimuli close to the hand. Subsequent studies confirmed that this visuo-tactile integration was not specific of PPS around the hand but could also be reported around other body parts, such as the face (Làdavas et al., 1998b; Farnè and Làdavas, 2002; Farnè et al., 2005a). From a neuroanatomical point of view, studies have shown that brain lesions in frontal, temporal and parietal cortex in the right hemisphere are the most common regions leading to extinction (Mattingley et al., 1997; Driver and Vuilleumier, 2001; Farnè et al., 2005b; Vossel et al., 2011; Kamtchum-Tatuene et al., 2017), at locations considered as the human homologues of the monkey cortical regions involved in PPS processing and described above. In particular, this neurological disorder appears most often in patients with focal inferior parietal lesions. Lesions of the temporo-parietal junction (TPJ), a region crucially involved in self-processing, also induce a disruption of PPS processing (Blanke et al., 2002; Blanke, 2012). The monkey homologue of TPJ is uncertain. A recent fMRI study suggests that the monkey homologue of human TPJ could actually lie midway along the ventral temporal sulcus (Mars et al., 2013) at a location where face and body patches are identified (Perrett et al., 1992; Tsao et al., 2003, 2008; Tsao and Livingstone, 2008; Rushworth et al., 2013; Popivanov et al., 2014; Premereur et al., 2016) and where impact prediction to the body produces strong neuronal activations (Cléry et al., 2017).

### Behavioral Evidence for the Existence of PPS

The above clinical evidence in favor of the existence of a PPS system in the human brain is corroborated by behavioral studies in healthy participants (Spence et al., 2004; Macaluso and Maravita, 2010; Occelli et al., 2011). These studies showed that the modulation of tactile perception by visual or auditory stimuli is more pronounced when these are presented close, as compared to far, from the body. Neuroimaging studies using EEG (Sambo and Forster, 2008), TMS (Serino et al., 2011) and fMRI (Bremmer et al., 2001; Makin et al., 2007; Brozzoli et al., 2011, 2013; Gentile et al., 2011) demonstrated that multisensory representation of PPS occurs in both parietal and prefrontal areas (**Figure 1B**) where PPS neurons have been identified in the homologous macaque regions (for reviews, see Cléry et al., 2015b; di Pellegrino and Làdavas, 2015).

**FIGURE 2:**
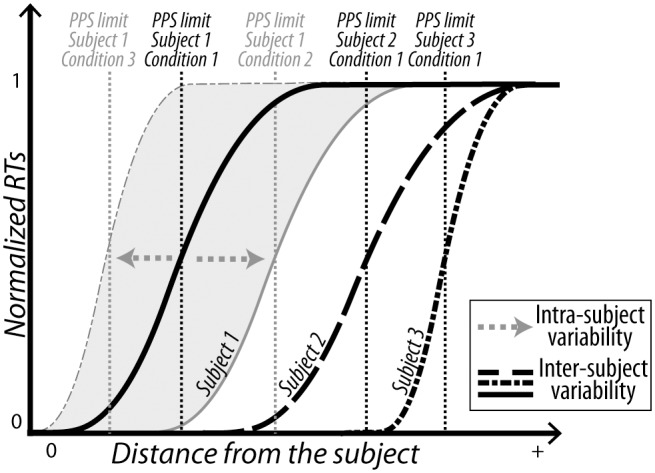
Intra and inter-individual variabilities for peripersonal space boundary. The limits between peripersonal space, closest to us, and far space, can vary within individuals as a function of sensory, cognitive or social context. These limits can also vary across individuals as a function of their own experiences and state (phobia, type of social interaction, etc.).

There is no physical separation between PPS (near space) and the extrapersonal space (far space) in the real world, however, the brain does represent, at least as assessed behaviorally, a boundary between these two spaces. That is to say between what is close to our bodies, which can potentially impact, interact with or attack us, and what is further away, at a distance that we cannot act upon except by a full displacement of the body. Importantly, this boundary is not fixed and can vary within and across individuals (Maravita and Iriki, 2004; Farnè et al., 2005a,b; Cléry et al., 2015b; de Vignemont and Iannetti, 2015). Indeed, the limits between PPS and far space can be very different from one subject to the other, as well as the sharpness of the representational gradient between these two spaces (**Figure 2**). Likewise, within a given subject, these limits can vary as a function of the sensory, cognitive or social context, and appears to be reliably skewed under certain psychiatric conditions (see for review Cléry et al., 2015b). Nevertheless, even if PPS can be modified in certain conditions, under specific controlled conditions and in a homogeneous sample (e.g., no phobia), it is possible to estimate PPS boundaries at least at group level.

### Possible PPS Functions

Objects approaching us or a predator may generate a threat or harm us, and induce the need to initiate defensive behavior. As a result, looming stimuli often indicate an intrusion or a risk of intrusion in our PPS. This correlates with an enhanced tactile processing as assessed both by d’-sensitivity measures and reaction time (RT) measures (Canzoneri et al., 2012; Cléry et al., 2015a; Kandula et al., 2015; De Paepe et al., 2016). As a result, PPS has been proposed to define a safety boundary around the body (Graziano and Cooke, 2006; Sambo and Iannetti, 2013; Cléry et al., 2015a,b, 2017, 2018; de Vignemont and Iannetti, 2015). However, PPS is also, by definition, the space that is close to our body, or self. Accordingly, recent studies and reviews highlight the link between PPS and body self-consciousness. For example, Grivaz et al. (2017) propose a meta-analysis of human studies, comparing the cortical bases of PPS and body self-consciousness, with a specific focus on their overlap and their respective specificities.

In the following, we will first review the different methods developed to measure PPS (see Measuring Peripersonal Space), the role of impact prediction in the definition of PPS (see Looming Stimuli and Touch or Impact Prediction to the Body), evidence for modulations of PPS (see Modulations of Peripersonal Space), a discussion on the modular nature of PPS (see Different Representations of Body-Related PPS) and last, the functional link between PPS and body self-consciousness (see Peripersonal Space and Bodily Self-Consciousness).

## Measuring Peripersonal Space

Both in the human brain and in the monkey brain, the neurons that represent PPS are more strongly driven by dynamic stimuli approaching the body than by static stimuli. This is for example the case for the bimodal and trimodal neurons that can be recorded both from the ventral intraparietal area (Colby et al., 1993; Duhamel et al., 1997) and the premotor cortex (Graziano et al., 1994, 1997, 1999; Fogassi et al., 1996). The firing rate of some of these neurons increases as function of the velocity of the looming stimulus, suggesting that these neurons might be computing the time to impact on the body (Fogassi et al., 1996). This is also observed behaviorally, as the velocity of looming audio stimuli has been recently shown to dynamically resize PPS (Noel et al., 2018a). This observation is suggested to be an emergent property of visuo-tactile recurrent neuronal networks proposed to mimic PPS parietal and prefrontal functions (Noel et al., 2018a). Looming stimuli have also been used to probe PPS in more complex designs. For example, Finisguerra et al. (2015) use TMS (transcranial magnetic stimulation) in order to quantify changes in hand cortico-motor excitability as a function of the position of a looming stimulus with respect to the subject’s hand.

Based on these findings, a method has been developed to estimate the boundary of PPS using dynamic stimuli. Indeed, these stimuli have a higher ecological relevance than static stimuli when it comes to studying PPS. Besides, this approach is more similar (though not identical) to the conditions used in monkey neurophysiology experiments, and thus makes it possible to directly compare the results across species (Canzoneri et al., 2012).

The idea behind this paradigm is to measure the behavioral responses in humans that are expected to reflect the properties and putative function of the RFs of PPS primate neurons. The paradigm relies on using a dynamic multisensory (audio-tactile or visuo-tactile) integration task in order to assess the limits of PPS (defined as the inflection point where a notable increase in multisensory integration can be observed) and is considered as a functionally and ecologically more relevant paradigm than previous designs. Specifically, participants have to respond as fast as possible to tactile stimuli presented somewhere on their body, while task-irrelevant heteromodal cues (auditory or visual stimuli) looming toward or receding from the body part stimulated by the tactile stimulus are presented (Canzoneri et al., 2012, 2013a,b, 2016; Teneggi et al., 2013; Galli et al., 2015; Noel et al., 2015a,b). On each trial, tactile stimuli are presented at different timing with respect to the trajectory of the sound/visual dynamic stimuli. In other words, the tactile stimulus is delivered when the sound or visual dynamic stimulus is perceived at a variable distance from the body of the subject. PPS limits is inferred from the function associating the measured RTs to the tactile stimulus at the body part of interest (the hand, the face or the trunk), to the distance at which the visual or auditory dynamic stimulus was presented.

Reaction times to tactile stimuli progressively slow down as a function of the distance at which the sound/visual looming stimulus is presented; and inversely, RTs progressively speed up as a function of the distance at which the sound/visual receding stimulus is presented. The authors propose that this function describes the link between tactile processing and the location of auditory or visual stimuli in space and allows to estimate the critical distance at which an external stimulus starts to affect tactile processing. This distance, along a spatial continuum between far space and the external surface of the body, allows to approximate the boundary of PPS representation in humans (**Figure 2**). In a recent study, we use a visuo-tactile version of this paradigm to demonstrate that PPS is not only characterized by a speeding up of RTs but also by an anticipated enhancement of tactile processing as assessed by changes tactile sensory d’ measures, in prediction of an impact to the body (Cléry et al., 2015a). We show that this enhanced tactile processing in anticipation of an impact to the body happens according to spatial and temporal coincidence laws very similar to those proposed to subserve multisensory integration processes (Stein and Meredith, 1993; Rowland and Stein, 2014).

This new paradigm was first developed and used in the context of a dynamic audio-tactile interaction task to investigate hand-related PPS thanks to tactile stimulations presented on the hand (Canzoneri et al., 2012, 2013a,b). This paradigm was also used to investigate the effect of social variables onto face-anchored PPS, using a dynamic audio-tactile interaction task with tactile stimulations delivered onto the face (Teneggi et al., 2013). Recently this paradigm was also adapted to studies investigating the full body illusion (Noel et al., 2015a,b; Serino et al., 2015b). More recently, this protocol was used to study and measures the spatial extend of human PPS in real virtual as well as in mixed realities environment. More complex version of this task are also under investigation, whereby three sensory modalities are used (visual, auditory and tactile) thus experimental approaching richer and more ecological sensory environments (Serino et al., 2018).

Overall, this paradigm opens new perspectives in the study PPS and how it is modulated by the context (top–down information, bottom–up evidence, social cues etc.), experience (learning, priors etc.) and action.

## Looming Stimuli and Touch or Impact Prediction to the Body

The ecological significance between static stimuli close to our body (e.g., a wall, a desk) and dynamic stimuli looming toward us (e.g., a mosquito, a ball) are different. Approaching stimuli are potentially more hazardous than other visual stimuli, even when they do not predict a direct impact to the body. A predator, a dominant conspecific, or a mere branch coming up at high speed are dangerous if one does not detect them fast enough to produce the appropriate escape motor repertoire. Such looming stimuli are known to trigger stereotyped defense responses (in monkeys: Schiff et al., 1962; in human infant: Ball and Tronick, 1971). Interestingly, looming stimuli which are explicitly threatening are perceived as having a shorter time-to-impact latency in comparison to objects moving at the same objective speed and which are not threatening (Vagnoni et al., 2012). This underestimation of approaching stimuli is also influenced by ones motor abilities, and is for example increased if subjects have their heads constrained by a chin rest compared to when standing freely (Vagnoni et al., 2017), the former condition possibly indicating, due to the constraint, an increased threat relative to the unconstrained condition. The neuronal underlying of this observation is to our knowledge, completely unexplored.

### Temporal Prediction

In a visuo-auditory context, looming visual stimuli have been shown to generate evident orienting behavior toward simultaneous and congruent auditory cues compared with receding stimuli, both in 5-month-old human infants (Walker-Andrews and Lennon, 1985) and in non-human primates (Maier et al., 2004). Looming structured sounds can specifically benefit visual orientation sensitivity (Romei et al., 2009; Leo et al., 2011). In a recent study (Cléry et al., 2015a), we show that subjects have an enhanced tactile sensitivity in the presence of looming visual stimuli as compared to receding visual stimuli, confirming the idea that looming stimuli are more relevant than receding stimuli to the body, and trigger enhanced and anticipated tactile processes. Indeed, while both size and depth cues most likely participate to the tactile sensitivity modulation on the face, this study indicates that the movement vector cue (away from or toward the subject) is the main cue affecting tactile detection. Indeed, slower looming stimuli lead to a delayed predicted time of impact on the face, and consequently to a delayed time at which tactile sensitivity is maximally improved (Cléry et al., 2015a). In other words, the trajectory and speed of the looming visual stimuli fully account for the temporal and dynamic predictive cues that are exploited by the brain to anticipate touch or impact to the body (Cléry et al., 2015a; Huang et al., 2018). Likewise, other auditory or visuo-tactile integration studies (Canzoneri et al., 2012; Kandula et al., 2015) have shown that RTs are shorter when a tactile stimulus is delivered at the impact time of the looming stimulus and suggest that looming stimuli predictively speed up tactile processing. Specifically, the speed of the looming stimulus seems to guide the nervous system in defining a high touch/impact probability window not unlike the multisensory temporal binding window described during the physiological and perceptual binding of two stimuli into the representation of a same and single external source and defining the degree of temporal tolerance of the brain in this binding process (De Paepe et al., 2016; Noel et al., 2016, 2018b; for review, see Wallace and Stevenson, 2014).

In this context, it is suggested that a visual stimulus looming onto the body and predicting an impact with a tactile stimulation onto the skin can be used to recalibrate PPS representation in an anticipated manner. A recent modeling study captures this idea whereby the training of a recurrent neural network results in a prediction of the anticipated tactile stimulation, the prediction error increasing with the distance of the visual stimulus from the skin, and the confidence of the prediction decreasing with distance (Straka and Hoffmann, 2017).

Overall, an enhanced processing of time to collision to the body can thus be observed and modeled within PPS. However, this might actually reflect a general enhancement in the processing of time to collision in general. Indeed, the prediction of collision between two objects placed within PPS appears to be extremely dependent onto temporal variations (e.g, differences in object velocities, Iachini et al., 2017). This possibly suggests an adaptive function of PPS to anticipate and prepare the appropriate overt behavior in response to external events happening within PPS, whether interacting with the body or not (Iachini et al., 2017).

### Spatial Prediction

Besides, we found that tactile d’, a direct measure of sensitivity, are improved not only at the predicted time but also at the predicted location of impact of a approaching visual stimulus to the face (Cléry et al., 2015a), fully mirroring the expected subjective consequences of the visual stimulus onto the tactile modality. This observation is suggested to be an emergent property of visuo-tactile recurrent neuronal networks proposed to mimic PPS parietal and prefrontal functions (Noel et al., 2018a). Importantly, this enhancement is also observed for stimuli trajectories that do not predict a direct impact to the face but rather brush past it, suggesting that the prediction of intrusion of a visual stimulus into PPS triggers the same tactile enhancement mechanisms whether a direct touch/impact on the body is actually expected or “just” an intrusion in PPS.

### Possible Neural Mechanisms

In addition to a baseline multisensory enhancement, tactile sensitivity thus appears to be further improved by the predictive components of the heteromodal auditory or visual stimuli. By definition, this process involves cross-modal influences, and it was suggested that the cortical regions processing this multisensory touch/impact prediction mostly overlap with the corresponding multisensory integration convergence and integration functional network. While this has never been explicitly investigated in these terms, early observations are in full agreement with this hypothesis. The visual response observed in parietal tactile neurons was first interpreted as an “anticipatory activation,” predicting touch in the matching skin (Hyvärinen and Poranen, 1974). Second, some neurons in the ventral intraparietal area (VIP) integrate vestibular proprioceptive self-motions and visual motion cues to encode relative self-motion relative to the environment (Bremmer et al., 1997, 2000, 2002a,b; Duhamel et al., 1997). In the same lines, vestibular inputs are shown to dynamically influence the multisensory PPS boundary and spatial self-representations in humans (Pfeiffer et al., 2018). These neurons have been shown to be activated by both visual and tactile stimuli (Duhamel et al., 1997; Guipponi et al., 2013, 2015) and show non-linear sub-, super-, or additive multisensory integration operations (Avillac et al., 2004, 2007). Recently, an fMRI study in the non-human primate confirms that this area VIP is involved in impact prediction to the face in a visuo-tactile context (Cléry et al., 2015b, 2017). As a result, this area appears to process both the consequences of ones’ own whole-body movements onto the environment as well as the consequences of movement of objects within the environment, relative to the body. Last, premotor area F4, an area highly connected with parietal area VIP, is also robustly activated, bilaterally by impact prediction (Cléry et al., 2015b, 2017). Most importantly, in both parietal area VIP and premotor area F4, these activations are systematically significantly larger when the approaching stimulus is spatially and temporally predictive of the tactile stimulus than when these two stimuli are presented at the same time, strongly suggesting that these two areas are indeed, at the neuronal level predictively processing temporal and spatial cues, possibly via non-linear integrative neuronal mechanisms (Cléry et al., 2015b, 2017).

As seen in Section “Peripersonal Space,” areas VIP and F4 are proposed to play a key role in the definition of PPS. In a recent monkey fMRI study we assess the neural bases of near and far space coding during naturalistic 3D moving objects (Cléry et al., 2018). This study clearly confirms the involvement of both VIP and F4 for PPS encoding (Cléry et al., 2015b:Figures 1B,C, 3; Cléry et al., 2018: Figures 4, 8). This confirms the prior observations from single neuron studies in monkeys (Rizzolatti et al., 1981b; Colby et al., 1993; Graziano et al., 1997; Bremmer et al., 2002a,b, 2013). However, two important observations need to be highlighted at this point. First, our fMRI data show that within an area VIP anatomically defined as the fundal intraparietal sulcus region (IPS), and functionally identified as the cortical region activated by large field visual stimulation (Colby et al., 1993; Bremmer et al., 2002a,b; Guipponi et al., 2013), only a small portion is activated by visuo-tactile convergence (Guipponi et al., 2013: Figure 5; Cléry et al., 2015b: Figures 2B, 3), impact prediction to the face (Cléry et al., 2015b: Figure 3B; Cléry et al., 2017: Figure 3) and near space processing (Cléry et al., 2015b: Figures 1B, 3; Cléry et al., 2018: Figures 4, 8). Importantly, the very same voxels are activated by visuo-tactile convergence, prediction of touch/impact to the body and selective near space encoding, suggesting that these different functions are possibly implemented by unique neuronal computations (see Cléry et al., 2015b, for discussion).

This set of monkey fMRI studies also allows to identify the larger cortical network involved in touch/impact prediction to the body and near space processing, encompassing, in addition to subsectors of the classically defined VIP, a subsector of premotor area F4, corresponding to the polysensory zone Pz, as well as the fundus of superior temporal sulcus FST and early striate and extra-striate areas. This extremely strong overlap between the touch/impact prediction to the body network and the near space processing network provides strong support to the idea that functionally, PPS includes the skin as a frontier of self, or alternatively, that the frontier of self is defined not only by the skin but also by PPS (these two views being functionally speaking, equivalent).

**FIGURE 3:**
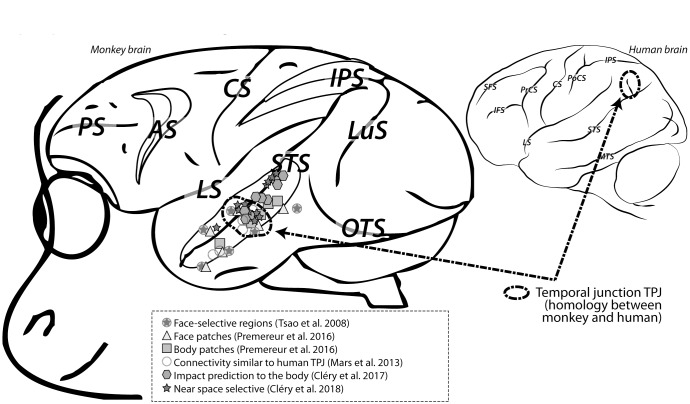
Functional overlap between temporal cortex regions involved in face processing (face patches), body processing (body patches), impact prediction and peripersonal space coding in the monkey brain. These overlapping regions are suggested to correspond to the monkey homologous regions of the human temporal junction TPJ. For other conventions, see **Figure 1**.

In **Figure 1**, a very good agreement can be seen between the premotor and intraparietal human and monkey PPS regions of interest (ROIs), as identified from a meta-analysis of the literature. In contrast, the monkey homologue of the human specific TPJ PPS ROIs, are not described. In a recent study based on the analysis of functional connectivity patterns, Mars et al. (2013) propose that the monkey homologue of human TPJ actually lies within the superior temporal cortex, at a location often associated with the processing of faces and other social stimuli (Perrett et al., 1992; Tsao et al., 2003, 2008; Tsao and Livingstone, 2008; Rushworth et al., 2013; Popivanov et al., 2014; Premereur et al., 2016). Importantly, this same region is found to be activated in our impact prediction to the face study (Cléry et al., 2017), as well as by objects looming toward PPS (Cléry et al., 2018), or placed within PPS (Cléry et al., 2018). **Figure 3** captures this functional overlap. As a result, we propose to expand the functions of this monkey STS region beyond the perception of faces and bodies to the processing of PPS in relation with one’s own body, homologous to one of the multiple functions of human TPJ.

### A Putative Defense PPS

A visual stimulus entering the PPS close to one’s cheek enhances tactile processing on that cheek, more than a visual stimulus which predicts an impact to the other cheek (Cléry et al., 2015a). This suggests that intrusion into PPS predicts touch or impact to the close by body surface. Canzoneri et al. (2012) demonstrate that the presence of a looming sound predicting an impact on the hand or within a well-defined distance from the hand, i.e., within a hand-referenced PPS, accelerates tactile processing on this hand. In monkeys, the electrical microstimulation of the neurons of these two regions induces a behavioral defense and avoidance repertoire of the entire body movements, indicating that they are involved in the coding of a defense PPS (Graziano et al., 2002; Cooke and Graziano, 2004; Graziano and Cooke, 2006). The size of this defensive space increases as the velocity of a potentially dangerous stimulus approaching the face increases (Bisio et al., 2017). Likewise, the size of PPS also increases as the probability that the looming threat stimulus impacts and harms the face increases (Bufacchi, 2017). All this taken together suggests the existence of a dynamic security margin around the face and the body.

One aspect of somato-sensation is nociception. In two studies, De Paepe et al. (2014, 2015) used temporal order judgment tasks, to assess whether the perception of nociceptive stimuli and their localization was influenced by proximal visual stimuli thus contributing to the construction of an integrated representation of PPS as has been described for touch. Participants were requested to judge which of two nociceptive stimuli was presented first, each stimulus being presented on one hand –the two hands being thus stimulated. Each dual nociceptive stimulation was preceded by visual cues presented either unilaterally or bilaterally, and either close to the subject’s body, or far from it. The authors further requested the participants to either cross their hands over their body’s midline or not. They found that the unilateral visual cue prioritized the processing of nociceptive stimuli delivered on the hand adjacent to the unilateral visual cue. This effect increased when the cue was displayed near to the participant’s hand (De Paepe et al., 2014), irrespective of posture. This demonstrates that the visuo-nociceptive interactions occur in a predominantly hand-anchored frame of reference and not in a body-anchored frame of reference and predominantly in a hand-anchored PPS (De Paepe et al., 2015; Filbrich et al., 2017). In a third study (De Paepe et al., 2016), participants were required to answer as fast as possible to indicate on which side they felt the nociceptive stimulus on their hand while a visual stimulus with different temporal onset synchronies was either looming or withdrawing with respect to the left or right hand of the participants. RTs were fastest when the visual stimulus was close to the stimulated hand and was more pronounced for visual looming stimuli. Taken together, these three studies confirm an interaction between the coding of nociceptive information and a peripersonal frame of reference bringing additional support to the proposal that PPS may contribute to the definition of a safety margin representation around us and having as a goal to keep us safe from any potential physical danger.

A recent review (Van der Stoep et al., 2015) suggests that, depending on their distance to the body, different combinations of sensory information might be more or less relevant. For example, touch and vision interactions are expected to dominate in PPS, as they correlate with an interaction between the body and the environment (e.g., for grasping or defense). In contrast, auditory and visual information may be more relevant in extrapersonal space away from the subject’s body as they provide information about far away objects, and contribute to spatial orienting, navigation and interaction with others (e.g., during conversation). As tactile stimuli can only be processed when applied to the body, audiotactile and visuotactile interactions (e.g., in the case of touch or impact to the body) by definition take place close to the body and PPS margin can thus be rationalized as the spatial alignment of different stimulus modalities with respect to the body. A more recent review from the same group (Van der Stoep et al., 2016) focuses on whether multisensory integration follows the same rules throughout the whole of 3-D space. Their meta-analysis highlights the fact that the region of space in which stimuli are displayed in, e.g., the distance to the body, modulates multisensory interactions, and that the space around us is separated into specific functional regions, defined by the body part they are mostly related to (e.g., the hand, the face or the trunk). Futures studies on PPS and notably on impact prediction onto the body need to take into account the several spatial constraints that are expected to influence multisensory integration processing: the spatial and temporal dynamics of the stimuli, the distance from the different body parts, the incidence of looming trajectories with respect to the body, the effects of body posture, the ongoing or planned movement of the subject as well as the social, valence and sensory nature of the environment and its organization with respect to the subject.

## Modulations of Peripersonal Space

Peripersonal space appears to have a singular function in our representation of space, associated, as described above, with an enhanced processing of sensory information as assessed behaviourally (RTs, sensory sensitivity) or functionally (single cell recordings, fMRI). In the last years, there has been a growing interest in the flexibility and plasticity of PPS (for review, see Cléry et al., 2015b; de Vignemont and Iannetti, 2015; Chris Dijkerman, 2017).

### Early Evidence for a Tool-Induced Reorganization of PPS

Several studies show that the use of a tool to reach objects in far space can extend the limits of PPS representation. In non-human primates, Iriki et al. (1996) demonstrated that, after training on the manipulation of a rake to access reward located at a distance beyond arm reach, hand-centered visual RFs of intraparietal neurons enlarged so as to encompass the rake. In humans, neuropsychological (Farnè and Làdavas, 2000; Maravita et al., 2001) and psychophysical (Holmes et al., 2004; Maravita and Iriki, 2004; Serino et al., 2007; Galli et al., 2015) studies showed that, after manipulating a tool, cross-modal interactions between visual or auditory stimuli presented in the far space and tactile stimuli at the hand increase. This is all the more pronounced at the location where the tool has been used. Taken together, these results bring support to the idea that the extent of PPS representation is dynamically reshaped by repeated experience and learning, allowing for an extension of the domain of action of the body beyond its structural limits (Maravita and Iriki, 2004; Gallese and Sinigaglia, 2010; Costantini et al., 2011). Early studies on this topic suggest that an active use of the tool is necessary for extending PPS representation. Persistence use, like in professional athletes (e.g., tennis players) or persons with disabilities (e.g., blind cane users), leads to a long-lasting incorporation of the tool into PPS even in the absence of the manipulation of the tool (Serino et al., 2007; Biggio et al., 2017). Last, tool-induced PPS plasticity is observed whether the tool is in physical interaction with the body (hammer, rack etc.) or not (mouse cursor, remote control of a sensory stimulus in far space etc., Goldenberg and Iriki, 2007; Bassolino et al., 2010; Serino et al., 2015a) indicating complex interactions between body schema and PPS for action. The immobilization of the right arm during 10h reduced PPS representation around this arm but without affecting the metric representation whereas the overuse of the left arm affected the metric representation but not PPS representation of this overused arm (Bassolino et al., 2015). This confirms the complex interactions between the body schema and PPS which are behaviourally dissociated.

### Sensory Synchrony as a Possible Trigger of Tool-Induced Reorganization of PPS

Serino et al. (2015a) propose the alternative hypothesis, that dynamic re-organization of PPS might from the integration of the experienced sensory feedback. Specifically, using a recurrent neural network model mimicking parietal multisensory neuronal organization, they show that the plasticity of PPS representation following tool-use arises neither from the function of the tool nor from the actions performed when using it, but is rather triggered by the experienced sensory feedback, i.e., the synchronous tactile stimulation of the hand when holding the tool and the heteromodal (auditory or visual) stimulation in the far space where the tool is being manipulated (for a review on tool-use, see Martel et al., 2016). In other words, temporal synchrony between (auditory or visual) sensory inputs in far space and tactile input arising from object manipulation by the hand in near space is suggested to have a major role in the functional definition of PPS from an action driven perspective. In a recent study (Cléry et al., 2018), we show that large cortical sectors are activates both by near and far space stimulations. We propose that these depth “non-specific” functional regions might support these dynamic associative mechanisms between far space and near space sensory stimulations.

### Non-motor Driven-Reorganization of PPS

Several studies show that tool use induce a remapping of PPS. This defines PPS from the point of view of a “goal-directed action” perspective in which we want to reach for something and grasp it (for review, see de Vignemont and Iannetti, 2015). However, recent evidence show that other cognitive factors than actions can remap this space such as fear, anxiety, social engagement and contribute to a “protective and defensive” view of PPS. These are reviewed below.

#### Bottom–Up Driven Reorganization of PPS

It is now well established that certain categories of bottom-up signals drive an instantaneous resizing of PPS. This is the case of threatening stimuli. For example, tactile processing is facilitated when physically threatening pictures (for instance a snake or a knife) are presented in PPS, generating to quicker responses than when such pictures are displayed in far space (Poliakoff et al., 2007; Van Damme et al., 2009). Likewise, sounds that elicit a negative emotion (e.g., screaming woman) or sounds that have a negative ecological connotation (e.g., barking dog), induce faster reactions times when they appear close to the subject as compared to neutral or positive valence sounds (Taffou and Viaud-Delmon, 2014; Ferri et al., 2015). In addition, the distance from a visual stimulus to the body has a stronger influence on RTs to a tactile stimulus on the skin if it is perceived as threatening. This indicates that not only PPS is resized by a threatening object, but the information relative to its distance from the body is enhanced relative to that of a non-threatening one (de Haan et al., 2016).

Importantly, whatever the estimated level of threat represented by a visual object, the observed expansion of PPS is reduced when the threatening part of dangerous objects is oriented toward participants, as compared to when oriented away (Coello et al., 2012). This suggests that the interpretation of the higher order context in reference with the body is crucial in affecting the boundary of PPS. In other words, the resizing of PPS is due both to bottom–up and top–down factors. All taken together, these different studies show that the emotional aspects and characteristics of the threating relation to the body influence the defensive PPS and the safety body margin. Quite surprisingly, the neural bases of these observations and the functional networks they involve are unknown to date.

#### Top–Down Driven Reorganization of PPS: Social Factors

Top–down factors are also shown to resize PPS. For example, the presence of an observer and the nature of the interaction with her/him reshape PPS representation (Teneggi et al., 2013). Indeed, PPS boundaries shrink when a neutral observer is standing in far space. This is not observed when the observer is replaced by a mannequin. This thus suggests that one’s PPS resizes in the presence of conspecifics. Importantly, this resizing depends on the nature of the social interaction with these observers. For example, PPS boundaries between self and an observer merge (i.e., expand) after an economic game with this person, but only if this person has behaved cooperatively (Teneggi et al., 2013). PPS is thus shaped by our valuation of other people’s behavior and is modulated by social interactions. A recent study (Pellencin et al., 2017) shows that not only the nature of social interactions (as constructed on the basis of past experience and information) but also the first impression of the person facing us, i.e., our social perception about this person (on the bases of immediate “bottom–up” perceptual cues: appearance, size, facial features, age, body posture etc.) affects our own multisensory PPS representation. This thus reflects a modulation of low-level 3D visual information processing by high-level cognitive variables and both automatic and constructed social cues.

The extension and shrinkage of our PPS representation may not be the only change triggered by the presence of others. Indeed, several studies suggest that the observation of sensory and motor experiences by others, whether humans or animals are remapped onto our own bodily representations, thanks to a so-called “mirror system” that has been described both in the monkey and human brain (Rizzolatti et al., 2001; Rizzolatti and Craighero, 2004; Sinigaglia and Rizzolatti, 2011; Rizzolatti and Fogassi, 2014; Rizzolatti and Sinigaglia, 2016; Rizzolatti and Rozzi, 2018). This system is activated both when we are touched onto our own body, when we view another person being touched, as well as when events occur in the space near the other’s body (Blakemore et al., 2005; Serino et al., 2008; Caggiano et al., 2009; Keysers and Gazzola, 2009; Cardini et al., 2010). Ishida et al. (2009), using single cell recordings in monkeys, show that bimodal parietal neurons which are activated by sensory events taking place in the space close to the monkey’s own hand also respond to events taking place in the space close to another monkey’s hand. Similar functional activations are observed in premotor cortex in humans (Brozzoli et al., 2013; Holt et al., 2014).

A review by Ishida et al. (2015) based on monkey neurophysiology as well as human fMRI studies, reports shared self-other body representation coding in multiple brain areas including visuo-tactile neurons in parietal cortex (Ishida et al., 2009), secondary somatosensory cortex (Keysers et al., 2004, 2010; Blakemore et al., 2005; Ebisch et al., 2008; Keysers and Gazzola, 2009) and in insular cortex (Fitzgibbon et al., 2010, 2012; Lamm and Singer, 2010; Krahé et al., 2013) associated with affective touch and interoception. Importantly, Maister et al. (2015) show that synchronous tactile stimulation on one’s own face and visual stimulation close to another person’s face results in a functional interaction between both PPSs, such that events taking place near to the other person’s face acquired improved the salience of stimuli occurring in one’s own PPS. Nicely complementing these observations, Teramoto (2018) shows that, detection of tactile stimulation onto one’s own hand is faster when a visual stimulus is approaching the hand of another person rather than when placed far away from this same person. All this brings support to the idea of shared inter-personal PPS representations. The underlying neuronal and network computations of this behavioral observation remain to be explored.

The discussion mostly addresses the effect of the presence of a conspecific onto PPS. However, more complex social factors might be at play, such as the location of others with respect to ourselves, as well as their orientation or inferred displacement coding trajectory. This would predict that the neural networks involved in the coding of self with respect to the environment, also code the spatial contingencies between oneself and others, possibly along a coding schema resembling what has been described in bat and rodent hippocampal neurons (Danjo et al., 2018; Omer et al., 2018).

#### Interactions Between an Action-Based Peripersonal Space and Interpersonal Space

Recent studies were interested in investigating the link between PPS for action, defined as the space around us and onto which we can act, and interpersonal space (InterPS), defined as the space in which we maintain a distance around our bodies and in which any intrusion by others may cause discomfort. As seen above, this space can be modified by emotional and socially relevant interactions, including complex social information such as perceived morality or cooperativeness of another person, age and gender (Iachini et al., 2015, 2016). PPS for acting and interpersonal space share a common motor nature and are sensitive, at different degrees, to social modulation. Hence the proposal that social processing might be embodied and grounded in the “body acting in space” (Iachini et al., 2014). The evidence in this respect is mitigated. Indeed, in the hands of Patané et al. (2016) tool-use remaps the action-related PPS, estimated by a reaching-distance toward another person, but does not alter the social-related interpersonal space estimated by a comfort-distance task. Besides, after a positive social interaction with another individual, the estimated intrapersonal space is reduced whereas, in the same time, the estimated PPS is extended, suggesting that these two space representations have no full functional overlap between them (Patané et al., 2017). In the same lines, the introduction of invisible body illusions results in dissociable changes in InterPS and PPS sizes (D’Angelo et al., 2017). In contrast, in the hands of Quesque et al. (2016), using a different paradigm in which participants observed a point-light walker approaching them from different directions and passing near them at different distances from their right or left shoulder, comfortable interpersonal distance, is found to be linked to the representation of PPS. This indicates that enlarging PPS through tool manipulation effect that comfortable interpersonal distance with respect to another person also enlarges, corroborating the hypothesis that interpersonal-comfort space and peripersonal-reaching space share a common motor nature (Iachini et al., 2014, 2016; Coello and Fischer, 2015). Further investigations will need to be performed in order to reconcile these two views.

#### Interaction Between PPS and Personality Traits

Peripersonal space size can be related to some key personality traits. The study of defensive reflex responses is instrumental to address this question. Indeed, these defensive reflex responses can be precisely adjusted by the location of the stimulus within PPS. An important aspect of this modulation in that it is specific to the body part for which the reflex response gives protection (Sambo et al., 2012a,b). For example, subcortical defensive responses like hand-blink reflex (HBR) are improved when a threat approaching the face by one’s own stimulated hand, by another person’s hand and when the hand of the participant enters in PPS of another person. Importantly, the interaction between these defensive reflexes vary from one individual to another, as a function of several personality traits. For example, the enhancement of the HBR is more important in participants with a strong empathic tendency when observing another person from a third person perspective, suggesting that interpersonal interactions modulate perception of threat and defensive responses and more so in empathic participants (Fossataro et al., 2016). Along the same lines, the size of an individual’s PPS is associated with trait anxiety, with an enlarged PPS in more anxious individuals (Sambo and Iannetti, 2013; for review, see de Vignemont and Iannetti, 2015). The passive listening to a conversation also affects the size of PPS/InterPS of a third person not involved in the conversation. Indeed, his/her PPS expanded if the conversation had an aggressive content compare to a neutral content, thus resulting in an increase in the peripersonal safety boundary in the face of a potentially aggressive confrontation (Vagnoni et al., 2018). Likewise, PPS size in claustrophobic subjects is different from that of non-claustrophobic subjects. Claustrophobia is a situational phobia characterized by intense anxiety in relation to enclosed spaces and physically restrictive situations (American Psychiatric Association, 2000). Lourenco et al. (2011) investigated whether the size of near space relates to individual differences in claustrophobic fear, as estimated from the reported anxiety in enclosed spaces and physically restrictive situations and show that claustrophobic fear is associated with an enlarged size of the close space directly around us. Vagnoni et al. (2012) show the same results and expand them by demonstrating that emotions, in addition to altering the perception of space as a static entity, also affects the perception of dynamically moving objects, such as those on a collision course with the observer. Importantly, claustrophobia is not only associated with an increased PPS relative to non–claustrophobic subjects, but it is also characterized by a less flexible PPS. Indeed, when using a stick during a line bisection task, whereas individuals low in claustrophobic fear demonstrate the expected expansion of PPS, individuals high in claustrophobic fear show less expansion following tool-use (Hunley et al., 2017).

In summary, PPS is not a fixed space but a dynamic space which is continuously modulated by our environment (social, emotional, functional). The dynamic adjustment of this “boundary” of self may be related to an optimization of the behavioral outcome and repertoire (protective, pro-active) to the outside environment, based on online estimation of bottom-up information (visual, tactile, auditory, proprioceptive…) as well as of top-down cognitive information (context, emotion, social interactions…) (Cléry et al., 2015b; de Vignemont and Iannetti, 2015). PPS can thus be viewed as the output computation of the integration of multiple sources of information dynamically linking the body with its environment. This predicts that the properties and specificities of PPS will depend on the body part it is referring to, including in the non-motor domains.

## Different Representations of Body-Related Pps

Most of studies on PPS targeted the hand and to a lesser extent on the face. We have seen that this “boundary” of PPS representation is modulated both by action (for example after tool-use) and emotional/social context (fear, anxiety, cooperation). Besides, these modulations can vary within individuals as a function of the context. A strong inter-individuals variation is also observed. The question we are addressing here is whether the representation of PPS follows the same constraints and rules for all body parts or not?

Measuring the influence of looming stimuli presented at different distances from a given body part on the RTs to a tactile stimulus (Canzoneri et al., 2012, 2013a,b; Teneggi et al., 2013; Galli et al., 2015; Noel et al., 2015a,b), Serino et al. (2015b) characterize PPS from a body-referenced perspective. In a first experiment, they test the effect of looming and receding auditory stimuli in relation to the trunk on tactile detection on this body part. As previously described for the hand and the face, they show that looming sounds modulate tactile processing depending on the distance of the sound from the body and that this effect is specific for looming sounds and is not observed for receding sounds. The majority of experiments on PPS are done only in the front space of the subject. Therefore, in a second experiment, the authors also introduce looming and receding auditory stimuli from the front or back of the peri-trunk PPS. They confirm that only sounds looming toward the trunk are mapped into the representation of the trunk-PPS. No notable difference can be observed between a frontal trunk-PPS and a hind trunk-PPS. In a third experiment, the authors test the effect of looming and receding auditory stimuli from the hand-PPS. They show that sounds modulate tactile processing according to the distance of the sound from the hand. This effect is observed not only for the looming sounds but also for the receding sounds, though the speeding of tactile detection on the hand is more pronounced for looming stimuli than for receding stimuli. Importantly, the distance at which the sounds started to have a significant effect onto tactile processing is shorter for the hand-PPS than for trunk-PPS, indicating that trunk-PPS is larger than the hand-PPS. The authors then confront the representations of the hand-PPS and trunk-PPS and how they interact. For this, while using looming and receding sounds from the stimulated body part, they apply tactile stimulations either to the trunk or to the hand placed close to the trunk (Experiment 4) or to the hand placed far from the trunk (experiment 5). The authors show that when the hand is close to the trunk, the trunk-PPS and its properties dominate onto the hand-PPS, while this is not the case when the hand is far away from the trunk. In summary, two different PPS representations can be distinguished, one anchored to the hand and that is sensitive to both looming and receding stimuli at close distance from the hand and another one, anchored to the trunk and sensitive only for looming stimuli and encompassing more PPS (in terms of distance to the body) than hand-PPS. Importantly, these two representations are not independent. To further investigate the nature of the interaction between sub-PPSs, the authors further test the effect of looming and receding stimuli (auditory or visual) from the trunk or the face PPS while tactile stimuli are presented either to the face or the trunk. Tactile processing on the trunk gets enhanced by looming stimuli both toward the face or the trunk, indicating that the trunk-PPS encompasses the face-PPS. The reverse is, however, not true, as tactile processing on the face is not enhanced by stimuli looming toward the trunk. Recently, the authors show that the velocity of looming auditory stimuli not only shape the peri-hand space, but also modulate the peri-face and the peri-trunk spaces (Noel et al., 2018a). They propose a neural network involving reciprocal connections between unisensory areas and higher-order multisensory neurons, with a neural adaptation to persistent stimulation, to account for these several behavioral observations characterizing PPS and its sub-PPS components (for details, see Serino et al., 2015a; Noel et al., 2018a).

**FIGURE 4:**
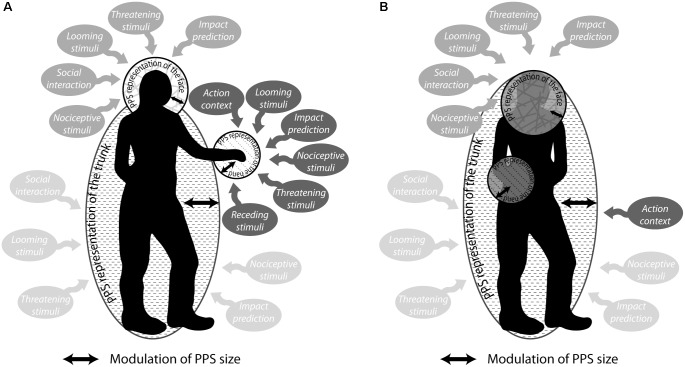
Peripersonal space representation is modulated by numerous factors such as impact prediction or social, emotional and action components. **(A)** There are at least three sub-representations of PPS: the trunk, the face and the hand (which can extend to incorporate lower limbs, Stone et al., 2017). **(B)** These representations can merge depending on their relative distance from the trunk.

To summarize this exhaustive study, Serino et al. (2015a) show that the size of PPS representation varies as a function of the stimulated body part, being gradually larger for the hand, the face and maximal for the trunk (**Figure 4A**). Tactile processing onto these specific body segments is modulated by looming stimuli, in a space-dependant manner. Most importantly, while the size of PPS representation around the trunk is relatively constant, PPS representation around the hand or the face vary according to their position relative to the rest of the body and relative to the trajectory of the stimulus relative to the body (**Figure 4B**). These observations are confirmed by more recent studies (Aggius-Vella et al., 2017) and also generalize to lower body segments (Stone et al., 2017). Indeed, Stone et al. observed that participants have speeded RTs to a tactile stimulus applied to the feet when a visual stimulus approaching the legs. In addition, they showed that, similar to what is observed for the hand, the leg is, in this condition, highly distorted (i.e., perceived to be wider or shorter than its actual physical dimension, Stone et al., 2018). These results are in agreement with the function of a PPS as a multisensory-motor interface for body-object interaction (Brozzoli et al., 2012b).

This first extensive mapping of humans PPS representation opens new perspectives in PPS research. For example, how are these body-part specific PPS representations incorporated in a “goal-directed action” or a “protective/defensive” view of PPS?

## Peripersonal Space and Bodily Self-Consciousness

The trunk-PPS representation integrates both body-related signals (proprioceptive, tactile) and information related to stimuli from the outside world (visual and auditory) that can potentially interact with the body, in a global, egocentric frame of reference. This representation may thus form a basic neural representation that is relevant for the definition of self, self-consciousness and self-consciousness in relation to the outside world (Tsakiris et al., 2007; Blanke and Metzinger, 2009; Tsakiris, 2010; Blanke, 2012; Blanke et al., 2015; Serino et al., 2015b). In the following, we will shortly review the growing evidence providing a possible link between PPS and self-consciousness.

Bodily self-consciousness (BSC), that is, the feeling that the physical body and its parts belongs to us (i.e., our own body), is proposed to be one of the main characteristics of subjective experience, i.e., binding whatever external or internal experience to self (Gallagher, 2000; Blanke and Metzinger, 2009). In the last years, multisensory bodily illusion paradigms have been used to investigate BSC in the laboratory, demonstrating, for example, the behavioral mechanisms underlying the perception of ownership of the hand using the rubber hand illusion (Botvinick and Cohen, 1998), or of the face using the enfacement illusion (Tsakiris, 2008; Sforza et al., 2010), or of the entire body using the full-body illusion, the out-of-body illusion or the body-swap illusion (Ehrsson et al., 2007; Lenggenhager et al., 2007; Petkova and Ehrsson, 2008). These illusions are based on the application of synchronous stimulations binding the body (or body part) of the participants, stimulated by touch, to a virtual body (or fake body part), stimulated visually. This type of experimental paradigms results in an illusory feeling of ownership toward the virtual body or body parts. These studies, have resulted in a general agreement that ownership over hands, face, and body in general, depends on the integration of multiple bodily signals in the brain, including tactile, proprioceptive, visual and auditory signals (Ehrsson et al., 2004; Makin et al., 2008; Tsakiris, 2010; Blanke, 2012; Ehrsson, 2012; Serino et al., 2013; Blanke et al., 2015). As a result, there seems to be a direct relationship between the neural mechanism underlying multisensory PPS processing and BSC. However, to date, these two processes and their underlying neuronal mechanisms were investigated separately. In a recent study, Grivaz et al. (2017), conduct an extensive meta-analysis of functional neuroimaging studies to find the key neural structures for PPS, for BSC and identify their possible functional overlaps in humans. The authors thus performed a systematic quantitative coordinate-based meta-analysis on human functional neuroimaging studies (Turkeltaub et al., 2002; Eickhoff et al., 2009, 2012). They selected 35 PET or fMRI studies: 18 studies assessing brain regions activated by the encoding of unisensory and multisensory stimuli within PPS (whether the hand, the face or the trunk PPS); 17 studies assessing brain regions activated by the BSC of the body or a part of the body. They identified a bilateral PPS network composed by superior parietal, temporo-parietal and ventral premotor regions. As discussed above, these regions play a key role in sensory-motor processes, mediating interactions between the subject and his/her direct environment, integrating sensory information and driving potential motor responses (Graziano and Cooke, 2006; Làdavas and Serino, 2008; Cléry et al., 2015b; Grivaz et al., 2017). On the other hand, the BSC network includes the posterior parietal cortex (IPS bilaterally), the superior parietal lobule (SPL), the right ventral premotor cortex, and the left anterior insula. These regions are involved in multisensory integration, attention and awareness. In particular, the insula plays a key role in the integration of exteroceptive body-related cues and interoceptive signals that are proposed to be crucial for subjective experience (Craig, 2009; Damasio and Meyer, 2009; Tsakiris, 2010; Seth, 2013; Park and Tallon-Baudry, 2014; Seth and Friston, 2016). Although BSC and PPS representations are not associated to the exact same functions, they do activate common fronto-parietal regions. Indeed, the conjunction analysis performed by Grivaz et al. (2017) shows that PPS and BSC tasks anatomically overlap in only two clusters located in the left parietal cortex (dorsally at the intersection between the SPL, the IPS and area 2, ventrally between area 2 and IPS). The activations of this dorsal SPL/IPS supports the hypothesis that multisensory integration of bodily cues contribute to the construction of both PPS and BSC (Brozzoli et al., 2012a; Gentile et al., 2013; Grivaz et al., 2017). A recent study by Salomon et al. (2017) shows that the integration of multisensory bodily inputs for PPS construction do not necessarily require conscious awareness while BSC, is by definition, a conscious process. This might correspond to a major hallmark differentiating these two processes.

Thus, overall, PPS and BSC are subserved by only partially overlapping functional networks supporting the idea that they correspond to two distinct functions, whereby PPS possibly implements a multisensory-motor interface for body-objects interaction and BSC is related with bodily awareness and self-consciousness. Importantly, in spite of the fact that they are not activated in PPS studies, the premotor and insular clusters implicated in BSC are systematically co-activated with the parietal clusters activated by PPS processing during numerous cognitive tasks suggesting that these regions are functionally interconnected.

## Conclusion

PPS representation is a complex psychological and functional construct that can be subdivided in multiple entities referenced to different body parts and whose exact configuration depend on multiple factors. This complex PPS representation continuously changes depending on the incoming bottom–up sensory information, motor experience e.g., during tool use, or top–down factors, including context, social interactions, personality or psychiatric traits (**Figure 4**). PPS representation is subserved by a well-identified parieto-temporo-frontal network that has some degree of overlap with the body self-consciousness network and one may predict that impairments in PPS representation or self-consciousness might have consequences on the other process. This opens new research directions for the future years.

## Author Contributions

JC and SBH outlined the review, wrote the manuscript, and designed the figures.

## Conflict of Interest Statement

The authors declare that the research was conducted in the absence of any commercial or financial relationships that could be construed as a potential conflict of interest.
